# Bibliographic research with large language model ChatGPT-4: instability, hallucinations and sometimes alerts

**DOI:** 10.1016/j.clinsp.2024.100409

**Published:** 2024-10-16

**Authors:** Konradin Metze, Rosana Celestina Morandin-Reis, Irene Lorand-Metze, João Batista Florindo

**Affiliations:** a Department of Pathology, Faculty of Medical Sciences, State University of Campinas, Brazil; b Faculty of Medical Sciences, State University of Campinas, Brazil; c Department of Internal Medicine, Faculty of Medical Sciences, State University of Campinas, Brazil; d Institute of Mathematics, State University of Campinas, Brazil

The public availability of large language models, such as ChatGPT, has increased their use in the area of medicine. Although this kind of artificial intelligence has been considered to be useful, there are also reports on disadvantages and even warnings of errors and incorrect information [1].

In the previous study with ChatGPT 3.5, it has been shown, that the retrieval of bibliographic data may show many inconsistencies and errors [2]. These findings inspired us to test an improved version of this LLM (ChatGPT-4) with a similar task. In the previous version 3.5, the authors observed that it was sometimes necessary to repeat a prompt up to seven times (on different days) until the system recognized bibliographic data.

Therefore, the authors investigated this question of temporal reproducibility now applying the ChatGPT-4 version, asking for information on professional and bibliographic data of all professors at a university department during a 10 day period. This task represents somehow a real-world situation: students or patients might be interested in the professional and scientific activities of the professors whose names they found in the home page of the University Hospital. Therefore, the authors submitted the following prompt every day during a ten-days-period (16‒25 October 2023).

“Please write a summary on the professional and scientific activities of (professor's full name) in the area of pathology or clinical pathology or hematology or neuropathology or microbiology or immunology or biochemistry or molecular mechanisms or cytology and add the most relevant literature, citing at least five references”.

The quality of the replies was checked by two observers comparing them with data from PUBMED, Web of Science, Google Scholar as well as Lattes database, which is an official platform of curricula supported by CNPq (National Council of Research) of the Federal Government.

References were classified as: a) Correct, b) With incorrect data but possibility of recovering the publication, or c) Hallucination with non-existing titles or existing publications written by others, but erroneously attributed to the person under investigation.

The descriptions of scientific and professional activities (sketches) were categorized by consensus discussion of two observers as: a) Acceptable, b) With deficiencies, and c) Insufficient (essential activities not mentioned or with relevant false information).

Bibliometric data of the 19 professors included in this study were retrieved from Clarivate's Web of Science (all databases used). Data ranged as follows: the number of publications (22‒343), citations (106‒8,289), and H-index (7‒48).

During the 10-day period, only eight professors had been recognized (between 2 and 10 times), Table 1. The authors found high Spearman correlation coefficients between the number of days when the person was recognized and the number of publications (*r* = 0.729; 95 % CI 0.461‒0.857; p < 0.001), citations (*r* = 0.702; 95 % CI 0.377‒0.868; p = 0.001), and the H-index (*r* = 0.741; 95 % CI 0.454‒0.882; p < 0.001), suggesting that the chatbot might find more easily data on researchers with a higher presence in the literature and academic reputation.

**Table 1.** Day-to-day responses of the ChatGPT-4 during a ten-day period.

Day	Person							
1	1	1	‒	0	1	2	2	0
2	‒	‒	‒	‒	1	2	2	2
3	1	1	‒	0*	‒	‒	2	1
4	2	0	0	0	‒	‒	0	2
5	2	0	0	0	‒	‒	2	2
6	2	0	0	‒	‒	‒	1	2
7	2	1	0	0*	‒	1	2	2
8	1	0*	0*	0*	‒	1	2	2
9	1	0	0	0	‒	‒	2	2
10	2	2	0*	0*	‒	2	2	2
Retrieved references	40	35	25	35	10	25	40	50
% of references with hallucinations	67.5	100.0	100.0	100.0	10.0	84.0	65.0	30.0

Eleven persons not recognized by the chatbot were not included. Important temporal instability of recognizing bibliographic and professional data of different professors. Variations in the quality of the descriptions of scientific and professional activities (0 = insufficient; 1 = with deficiencies; 2 = acceptable; * = hallucinated information). Please note the high percentage of hallucinated references. (‒) = Not recognized.

A total of 260 references were released (zero to five per reply), classified as follows: a) 11.54 % “Correct”, b) 13.85 % “With incorrect data” and c) 74.62 % as “Hallucinations”. The latter showed no repetitions, thus suggesting an influence of random processes during the creation.

Of the 60 sketches released, 41.67 % were “acceptable”, 21.67 % were “with deficiencies” and 36.67 % were “insufficient”. In seven sketches belonging to three professors, nonexistent information (“hallucinations”) was mixed up with true facts (Table 1). The quality of the sketches could vary considerably, alternating between “insufficient” and “acceptable” even in persons with a high H-index, sometimes on consecutive days. In certain cases, all references were hallucinated but the sketch was “acceptable”. The titles of the hallucinated references were sound in most of the cases, i.e., the content was perfectly aligned with the professor's activity and suggested some interesting actual investigation. A scientist confronted with them commented that these hallucinated titles were interesting, and that he would like to have performed these investigations. One possible explanation for this might be that, although, without access to real references, the chatbot might have created a stereotypic figure and also the topics of investigations which most probably these researchers might have performed.

Special attention should be drawn on the release of the second day, where only four professors had been recognized, three with acceptable sketches and one with deficiencies. All references however were hallucinated. Interestingly, also the following warning has been released for all cases: “Note: The above references are fictional and meant to represent the type of research one might expect based on the description of Dr. X's work. They are not actual publications by Dr. X)”.

Therefore, the authors can conclude that the system is somehow “aware” that the hallucinated references had been fabricated.

The problem is, however, that this warning was only released on one single day and therefore potential users would not have been warned on the other 9 days.

The present results suggest that the examined ChatGPT-4 version is not appropriate for bibliographic research, reinforcing the WHO warning that the actual versions of chatbots should not be considered suitable for recovering data from developing countries [3].

However, it could be more useful if combined with new advanced tools, such as Retrieval Augmented Generation (RAG) and prompt engineering. The authors also noticed that the limitations of LLMs are even more evident for researchers with lower publication and citation indexes. This is mostly associated with the limited representation of data in the training set for those cases. Such a scenario naturally suggests strategies to augment the training data providing some specific context for the task at hand. RAG is a well-studied technique for that purpose [4]. Techniques to encourage the LLM to say that it does not know the answer instead of hallucinating would also be valuable in this context. Nevertheless, the use of advanced strategies like that is still far from accessible for most of the users of LLMs, which conveys an impression that those models are not capable of fulfilling their promises and expectations in practice.

## Authors' contributions

KM: Design and planning of the study, critical review of the AI responses, data analysis, writing of the text and final approval of the text.

RM-R: Planning of the study, critical review of the AI responses, data collection and analysis, and final approval of the text.

ILM: Data analysis, organization of the study, drafting of the manuscript and final approval of the text.

JBF: Design and planning of the study, supervision of the data analysis, review and final approval of the text.

## Declaration of competing interest

The authors declare no conflicts of interest.
